# Prognostic value of histological subtype in intraductal papillary mucinous neoplasm of the pancreas: A retrospective analysis of outcome from one single center

**DOI:** 10.1097/MD.0000000000006599

**Published:** 2017-04-14

**Authors:** Yefei Rong, Dansong Wang, Chen Xu, Yuan Ji, Dayong Jin, Wenchuan Wu, Xuefeng Xu, Tiantao Kuang, Wenhui Lou

**Affiliations:** a Department of Pancreatic Surgery; b Department of Pathology, Zhongshan Hospital, Fudan University, Shanghai, China.

**Keywords:** histological subtype, IPMNs, prognosis

## Abstract

We sought to retrospectively analyze the outcomes of patients with intraductal papillary mucinous neoplasm (IPMN) at our pancreatic surgery center, and to evaluate the prognostic value of histological subtype.

The clinical data of 121 IPMNs treated in our center between 2005 and 2014 were retrospectively analyzed. Pathological slides were thoroughly reviewed by 2 specialized pathologists.

Of the 121 patients, 48, 57, and 16 had main-duct, branch-duct, and mixed type IPMNs, respectively. Forty-one patients had invasive IPMNs. Histological subtypes consisted of 35 intestinal (28.9%), 56 gastric (46.3%), 29 pancreatobiliary (24.0%), and 1 oncocytic type (0.8%). Histological subtype was associated with radiological type, T stage, and degree of dysplasia (*P* < .05). No significant difference in overall survival was observed among the 4 histological subtypes, regardless of whether we considered all IPMNs (*P* *=* .106), or invasive IPMNs only (*P* *=* .828). However, the overall survival was associated with radiological type, T stage, degree of dysplasia, lymph-node status, and nerve invasion. For invasive IPMNs, the overall survival was associated with nerve invasion and lymph-node status; however, the association between nerve invasion and overall survival lost statistical significance after multivariate analysis.

Histological subtype had limited prognostic value in patients with IPMNs, and the main prognostic factor for patients with invasive IPMNs was the lymph-node status.

## 1 Introduction

Intraductal papillary mucinous neoplasm (IPMN) was first reported in the 1980s^[1]^ and in 1996 the entity of IPMN was included in the World Health Organization (WHO) classification system.^[2]^ IPMN grows in the main duct or branch duct of pancreas, which could produce mucin (MUC) with intraductal papillary projections of tall columnar epithelieum, and without subepithelial ovarian-type stroma, distinguishing it from mucinous cystic neoplasm.^[3]^ IPMNs are classified into 4 categories depending on the degree of dysplasia: IPMN adenoma, borderline IPMN, IPMN with carcinoma in situ, and IPMN with invasive carcinoma. Except for the pancreatic intraepithelial neoplasia, IPMNs are the most important precursor of pancreatic ductal adenocarcinoma.^[4]^ Compared with noninvasive IPMN, invasive IPMN has worse prognosis, with a 5-year overall survival rate of 24% to 40%.^[5–7]^ Generally, the prognosis of invasive IPMN is associated with radiological type, size of cystic mass, presence of mural nodules, positive lymph nodes, and positive cystic fluid cytology.^[8]^

IPMNs are classified into the main duct and branch duct types according to the site of origin. Depending on the microscopic morphological characteristics, the IPMNs are distinguished as intestinal, pancreatobiliary, oncocytic, and gastric subtypes. The intestinal, pancreatobiliary, and oncocytic subtypes largely originate from the main duct, whereas the gastric subtype derives from the branch duct.^[8]^ Although some studies have evaluated the prognostic value of histological subtype on IPMNs, their results are controversial.^[9–14]^ Therefore, the aims of this retrospective study were to evaluate the prognostic value of the histological subtype on IPMNs and to analyze the outcomes of patients with IPMNs from a single institution in China, at which all of the patients received uniform treatment, including uniform surgical indications and standards for operative technique.

## 2 Materials and methods

### 2.1 Patient selection and data collection

In this retrospective study, we analyzed data from 121 patients with IPMNs who underwent surgical resection between 2005 and 2014. This study was approved by our institutional review board and all patients provided informed consents. Clinicopathological data and radiological images were collected.

All samples were reevaluated by a senior pathologist with specific expertise in pancreatic pathology. IPMNs were diagnosed based on microscopic morphology, and were divided into 4 subtypes according to the MUC expression profiles. The surgical indications for main-duct IPMNs and branch-duct IPMNs followed the Sendai Criteria: a cyst of greater than 3 cm, a main pancreatic duct dilation exceeding 5 mm and mural nodules.^[15]^

### 2.2 Radiological diagnosis

Main-duct IPMN was defined as having main pancreatic duct dilatation over 5 mm. Branch-duct IPMN was defined as having cystic dilatation of the branch pancreatic duct that had communication with the nondilated main pancreatic duct. Mixed-type IPMN had characteristics of both main duct-type and branch duct-type IPMN.

### 2.3 Pathological assessment of IPMN

**Figure 1:**
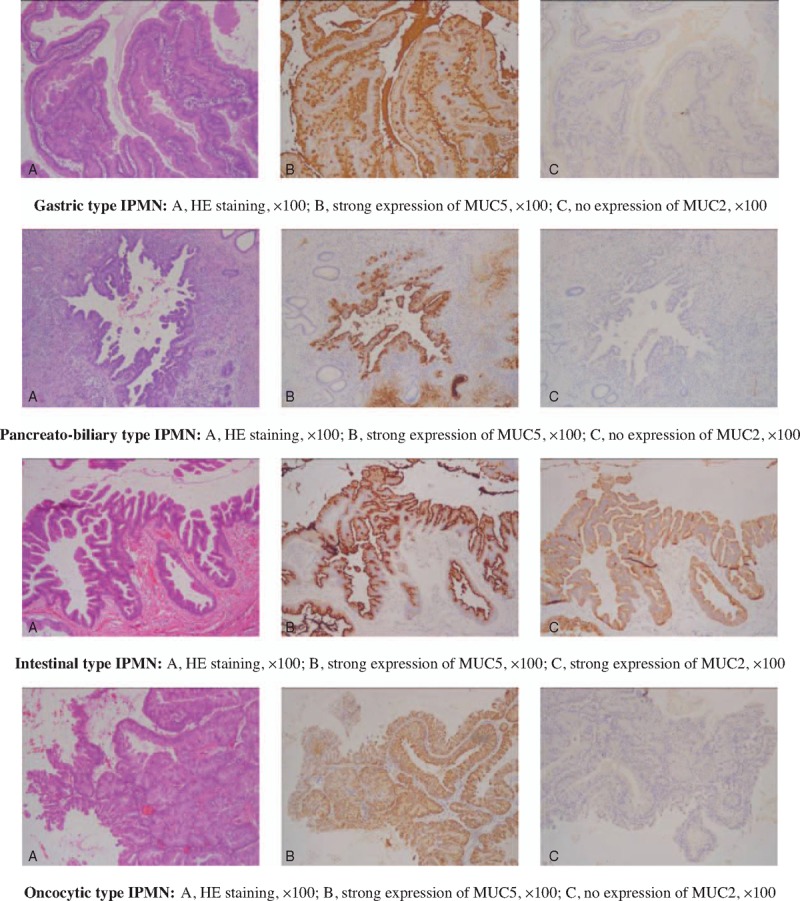
HE staining and immunohistochemical imaging of different IPMN subtypes. HE = hematoxylin and eosin, IPMN = intraductal papillary mucinous neoplasm.

The pathological assessments of IPMNs included the degree of dysplasia, lymph node metastasis, nerve invasion, and histological subtypes. The degree of dysplasia was categorized according to the WHO classification, which included the low-grade, intermediate-grade, and high-grade dysplasia and invasive IPMN. Histological subtypes were categorized as gastric, intestinal, oncocytic, or pancreatobiliary type, based on microscopic morphology with hematoxylin and eosin staining and immunohistochemical staining of MUC (Fig. 1). Tumor stage was recorded according to the 2002 TNM classification of the American Joint Committee on Cancer (AJCC).

### 2.4 Statistical analysis

SPSS (version 19.0, IBM Corp, Somers, NY) was used for the statistical analysis. The median survival times and the 95% confidence intervals (CIs) were calculated using the Kaplan–Meier method, and survival differences were analyzed with the log-rank test. Categorical data were analyzed using the *χ*^2^ test and analysis of variance. Factors that were significant (*P* *<* .05) at the univariate level were entered into the multivariate model. A Cox regression multivariate analysis with stepwise backward elimination based on the likelihood ratios was employed to test for independent predictors of the outcome. Because there were few cases of oncocytic-type IPMNs, *P* values among the gastric, intestinal, and pancreatobiliary types were also represented. *P* < .05 was considered to be statistically significant.

## 3 Results

### 3.1 Patients’ profiles

The patients’ profiles are presented in Table 1. The study cohort consisted of 78 man and 43 women with a mean age of 61.5 years at diagnosis (61.5 ± 9.8), and they were followed up for a median of 59.2 months (range, 4.3–126.9 months). Radiological type was identified as main duct type (n = 48, 39.7%), branch duct type (n = 57, 47.1%), and mixed type (n = 16, 13.2%).

**Table 1:**
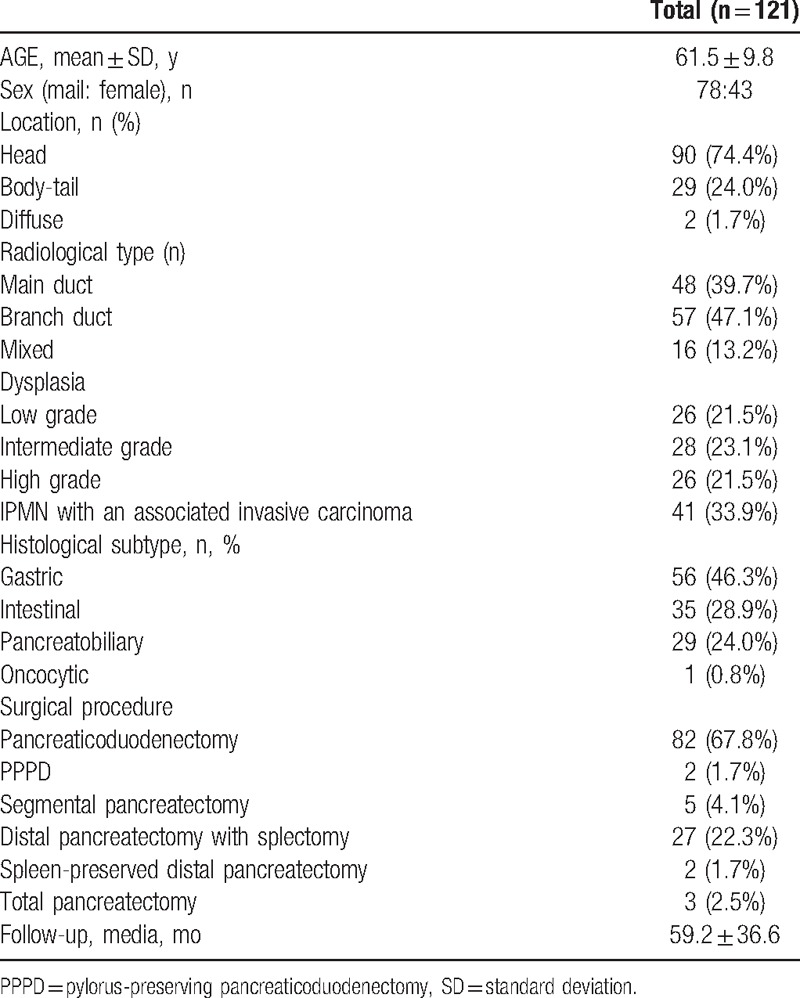
Patients’ demographics.

The surgical procedures included pancreaticoduodenectomy (n = 82), pylorus-preserving pancreaticoduodenectomy (PPPD, n = 2), segmental pancreatectomy (n = 5), distal pancreatectomy (n = 27), spleen-preserved distal pancreatectomy (n = 2), and total pancreatectomy (n = 3).

### 3.2 Clinicopathological characteristics

The histological subtypes of this study consisted of 56 gastric type (46.3%), 29 pancreatobiliary type (24.0%), 35 intestinal type (28.9%), and 1 oncocytic type (0.8%). The clinicopathological characteristics of the patients with IPMNs according to histological subtype are listed in Table 2. Histological subtype was not associated significantly with age (*P* = .636) or sex (*P* = .364). Regarding the radiological classification of IPMNs, the gastric subtype was more common among branch duct-type IPMNs, while the intestinal and pancreatobiliary types were more common among main duct and mixed duct-type IPMNs (*P* = .000). Gastric (69.0%) and intestinal (45.7%) subtypes were more frequently observed as low- or intermediate-grade dysplasia than was the pancreatobiliary type (17.2%, *P* = .004). Proportions of invasive IPMNs of each subtype were 25.0%, 25.7%, 62.1%, and 0 in gastric, intestinal, pancreatobiliary, and oncocytic types of IPMNs, respectively.

**Table 2:**
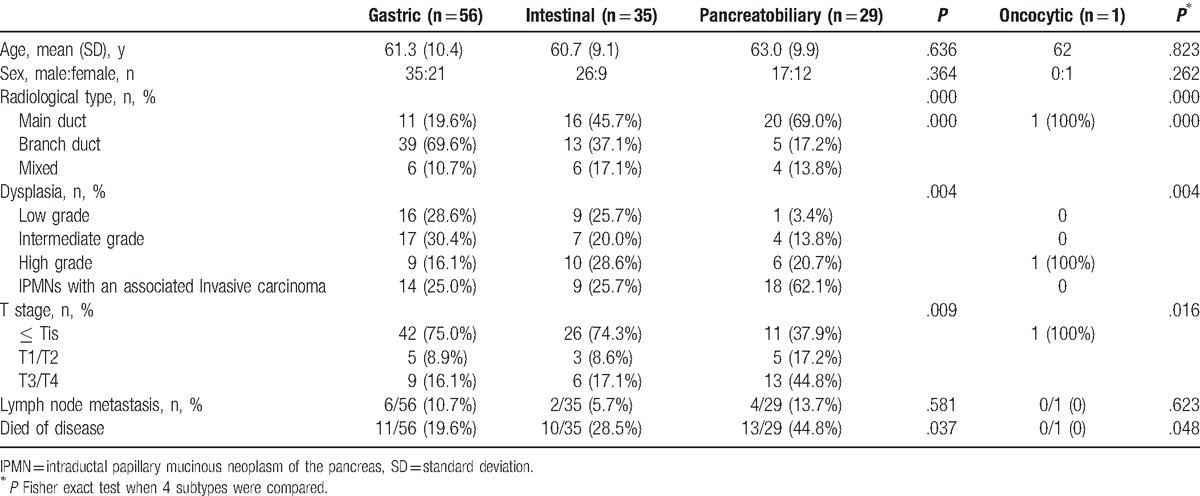
Clinicopathological characteristics of IPMNs according to histological subtypes.

Of all the patients, 41 patients (33.9%) had invasive IPMNs (Table 3). Among the patients with invasive IPMNs, the histological subtype did not show significant associations with age (*P* = .729), sex (*P* = .288), radiological type (*P* = .185), histology of invasive components (*P* = .819), T stage (*P* = .911), or lymph node metastasis (*P* = .415).

**Table 3:**
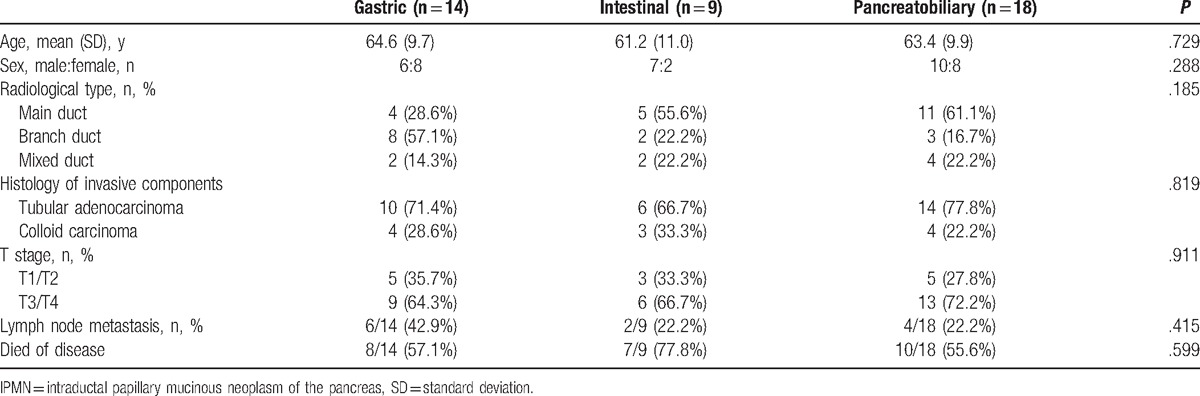
Clinicopathological characteristics of invasive IPMNs.

### 3.3 Survival analysis

Of the 121 patients in this study, 34 (28.1%) died of IPMNs, of which 25 (20.7%) cases’ death was found with invasive IPMNs.

**Figure 2:**
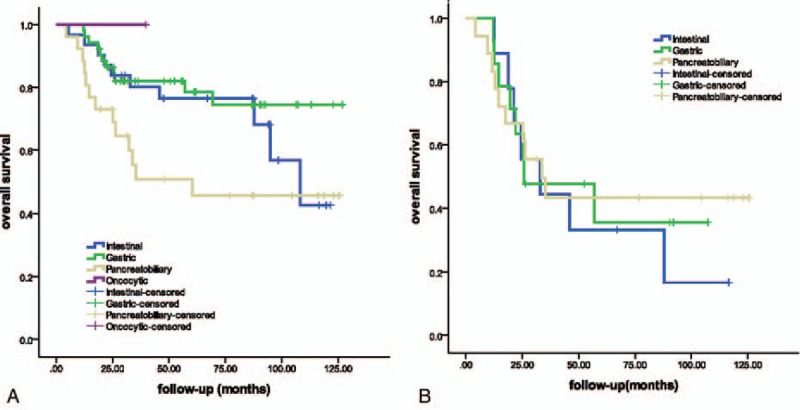
Overall survival curves according to the histological subtypes. A, Overall survival curves according to the histological subtypes (*P* = .106), and the 5-year survival rates were 78% (95% CI 66.2%–89.8%), 77% (95% CI 61.3%–92.6%), and 51% (95% CI 31.4%–70.6%) for gastric, intestinal, and pancreatobiliary subtypes, respectively. The overall survival of gastric subtype was better than that of pancreatobiliary subtype (*P* = .01). B, Overall survival curves of IPMNs with an associated invasive carcinoma according to the histological subtypes (*P* = .828). The 5-year survival rates were 36% (95% CI 8.6%–63.4%), 33% (95% CI 1.6%–64.4%), and 43% (95% CI 19.5%–66.5%) for gastric, intestinal, and pancreatobiliary subtypes, respectively. IPMN = intraductal papillary mucinous neoplasm.

The 5-year overall survival rate was 71% (95% confidence interval [CI], 61.2%–80.8%). According to the histological subtype, the 5-year overall survival rates were 78% (95% CI 66.2%–89.8%), 77% (95% CI 61.3%–92.6%), and 51% (95% CI 31.4%–70.6%) for the gastric, intestinal, and pancreatobiliary subtypes, respectively. The overall survival rates did not show a significant association with the histological subtype (*P* = .106, Fig. 2A). However, the overall survival of gastric type was much better than that of pancreatobiliary type (*P* = .01).

We also evaluated the prognositic value of histological subtype among the patients with invasive IPMNs. Among patients with invasive IPMNs, the histological subtype was not significantly associated with the overall survival (*P* = .828, Fig. 2B). There was no significant difference in 5-year survival rates between the gastric, intestinal, and pancreatobiliary subtypes (36% [95% CI 8.6%–63.4%]), 33% [95% CI 1.6%–64.4%], and 43% [95% CI 19.5%–66.5%], respectively).

Of all the patients, the degree of dysplasia (*P* = .000), T stage (*P* = .000), radiological types (*P* = .001), lymph-node status (*P* = .000), and nerve invasion (*P* = .000) were significantly associated with the overall survival (Table 4). Among the patients with invasive IPMNs, the lymph-node status (*P* = .001) and nerve invasion (*P* = .014) were significantly associated with the prognosis, while the T stage (*P* = .344), radiological type (*P* = .271), and histological subtypes (*P* = .828) had no significance concerned with the prognosis (Table 4). In addition, the multivariate Cox regression analysis for prognostic factors of invasive IPMNs revealed that only the lymph-node status (*P* = .013) was an independent prognostic factor (Table 5).

**Table 4:**
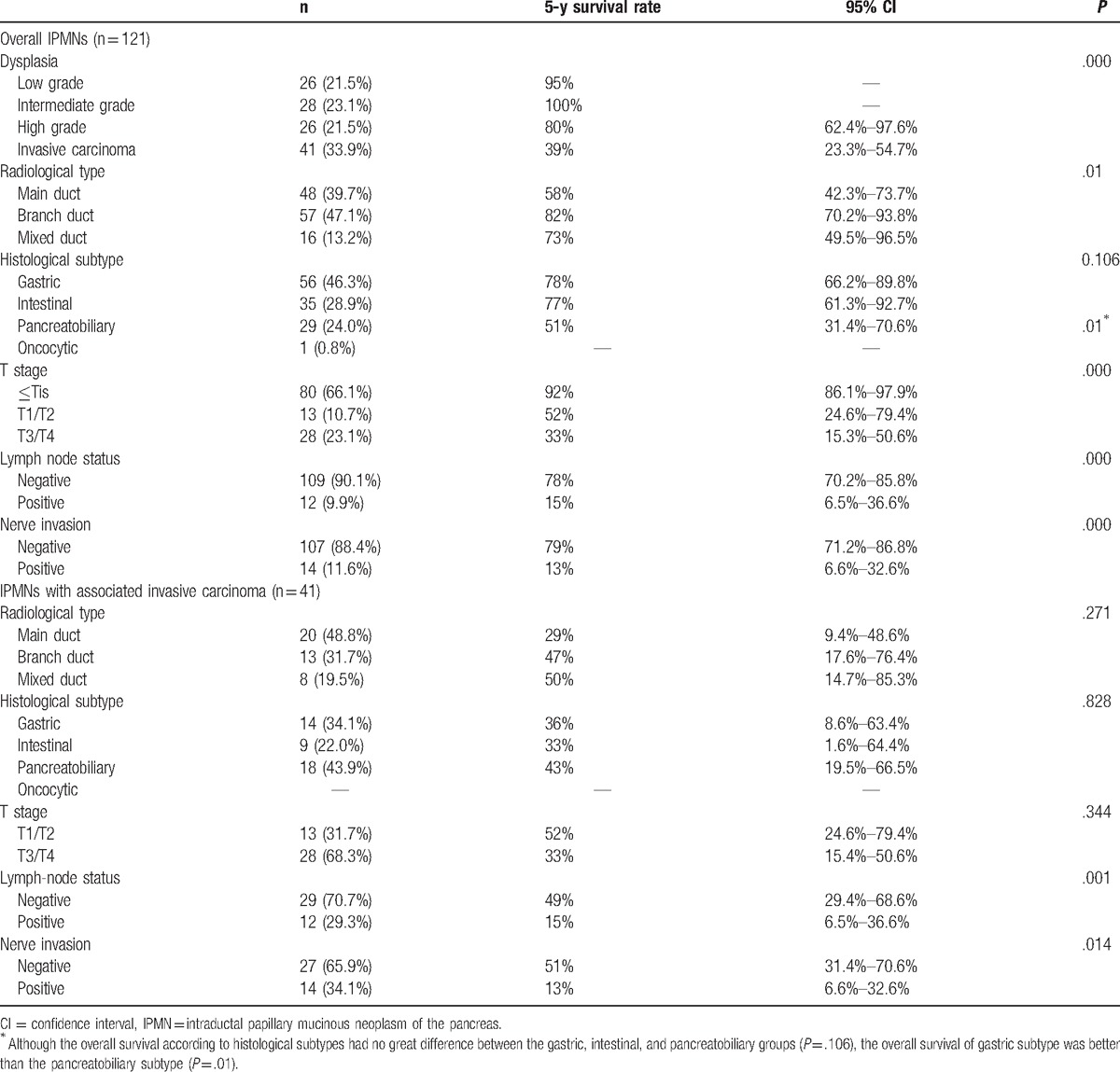
Analysis of prognostic factors of IPMNs.

**Table 5:**
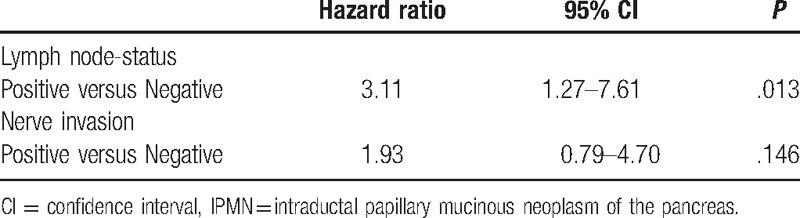
Cox proportional hazards model analysis for factors associated with survival of invasive IPMNs.

## 4 Discussion

Furukawa et al^[16]^ were the first to report a new classification system for IPMN that was based on the morphological phenotypes. This subclassification categorizes IPMNs into 4 subtypes: the intestinal, gastric, pancreatobiliary, and oncocytic type.^[16]^ However, the prognostic value of the histological subtype on IPMNs was controversial. Sadakari et al^[11]^ reported that the invasive carcinoma derived from the nonintestinal type had a poorer prognosis than that derived from the intestinal type and the 5-year survival rate of patients with the nonintestinal type was as poor as that of patients with conventional invasive ductal adenocarcinoma. However, the following articles had different results. Ishida et al^[13]^ reported that the gastric-type IPMNs had a better prognosis than the intestinal-type IPMNs, while Takasu et al^[14]^ reported that the gastric and intestinal type IPMNs had a comparable prognosis. In 2011, Furukawa et al^[17]^ demonstrated the significant prognostic relevance of these 4 subtypes with respect to disease-specific survival, which was highest in the gastric type and lowest in the pancreatobiliary type. Marius et al^[10]^ compared the prognosis of IPMNs of the 4 types and with that of the pancreatic ductal adenocarcinomas. Their results showed that the 5-year survival of patients with intestinal IPMNs was significantly better than that of patients with pancreatobiliary IPMNs. Further, the pancreatobiliary subtype was strongly associated with malignancy, and recurrence, and the overall survival of patients with this subtype was as poor as that of patients with pancreatic ductal adenocarcinoma.^[10]^ However, Mee et al^[9]^ reported that disease-specific survival was not associated with histological subtype in overall patients. For invasive IPMNs, histological subtype had a marginally significance on survival, which lost statistical significance after multivariate analysis. Their results suggested that the histological subtypes might have limited prognostic value for pancreatic IPMNs.^[9]^ Our results were consistent with their research.

In this study, the associations between histological subtype of IPMNs and radiological types or pathological characteristics were consistent with previous reports. In our study, the gastric subtypes were mainly derived from the branch duct (69.6%), while the pancreatobiliary subtypes were mainly derived from the main duct (69.0%). The gastric and intestinal subtypes had more low- or intermediate-grade dysplasia compared with the pancreatobiliary subtype (69.0%, and 45.7% vs 17.2%, *P* = .000). The overall survival times did not differ significantly among patients with these 4 subtypes; however when we limited the comparison to the gastric subtype and pancreatobiliary subtype, we observed that the overall survival of patients with the gastric type was much better than that of patients with pancreatobiliary type (*P* = .01, Fig. 2A). When analyzed among patients with invasive IPMNs, the overall survival lost its relationship with the histological subtypes and the 5-year survival rates did not differ significantly between the gastric, intestinal, and pancreatobiliary (*P* = .828, Fig. 2B).

Although we did not think that the prognostic value of histological subtype had been resolved by our study, the results that we observed had several useful implications. First, the fact that studies had small sample size was likely to be 1 of the main reasons that prognostic findings had been controversial. To date, the study population in the reported articles was mainly less than 200,^[9–14]^ and there were only 121 cases in the present study. Second, the composition of the patient cohort was different in each study. In Ishida's study, their study population only had 4 cases of pancreatobiliary subtype, while intestinal and gastric subtypes had 29 and 27 cases, respectively.^[13]^ While in Marius’ study, there were only 13 (12%) cases of gastric subtype of IPMNs, and the most populations were intestinal (44%) and pancreatobiliary types (40%).^[10]^ In our study, gastric type cases were most common with 46.3%, 28.9%, and 24.0% cases of gastric, intestinal, and pancreatobiliary subtypes, respectively. In addition, most of the patients in gastric subtype had low- or intermediate-grade dysplasia (69%), while most of the patients in pancreatobiliary type had invasive IPMNs (62.1%). Accordingly, there was no doubt that the overall survival of gastric subtype was much better than the pancreatobiliary type. However, once the gastric subtype transformed into invasive neoplasm, the overall survival would decrease dramatically. Previous studies had reported that when the gastric subtype transformed into malignant tumor, it mainly developed into invasive tubular adenocarcinoma that had much poorer prognosis than the invasive adenocarcinoma originating from the nongastric type.^[18,19]^ In addition, pancreatic ductal adenocarcinoma might mainly arise in the pancreas with benign gastric-type IPMN, in the absence of *GNAS* mutations.^[18,20]^ In our study, 71.4% of the gastric subtype invasive IPMNs were tubular adenocarcinoma, which was close to the pancreatobiliary subtype (77.8%) and the intestinal subtype (66.7%). The similar rates of tubular adenocarcinoma among invasive IPMNs of these 3 subtypes might explain the lack of any significant difference between the prognosis of patients with these subtypes.

Previous studies have reported that oncocytic type IPMNs accounted for 0.9% to 8.5%,^[9,10,17,18]^ whereas only 1 patient (0.8%) was diagnosed with the oncocytic subtype in our study. The prognosis of oncocytic type IPMNs was controversial also. Adsay et al^[21]^ reported 11 patients with oncocytic type IPMNs: 1 died of cancer, 3 died of other diseases, and 7 remained alive without recurrence. Takasu et al^[14]^ reported 3 patients with oncocytic tumors whose prognosis was significantly poorer than that of patients with gastric or intestinal type IPMNs. Marchegiani et al^[22]^ reported a large population of oncocytic IPMNs including 18 patients. The prognosis of these patients was excellent. At a median follow-up of 7 years, no patients with oncocytic IPMN had died from the disease.^[22]^ In our study, there was only 1 oncocytic IPMN with high grade dysplasia and this patient survived for 39 months after surgery until the follow-up deadline.

Previous researches have revealed that lymph node metastasis, vascular invasion, positive surgical margin, and tubular invasive pattern are associated with the poor prognosis in cases of the invasive IPMNs.^[8]^ In our study, the positive lymph-node status and nerve invasion were associated with the poor prognosis in patients with invasive IPMNs, and multivariate analysis demonstrated that the lymph-node status was a significant and independent prognostic factor (*P* = .013). However, the association between the nerve invasion and prognosis lost its statistical significance in the multivariate analysis (*P* = .146), which might be due to the small number of the invasive IPMNs in our study cohort. In addition, the lymph-node status did not show a significant association with the histological subtype of IPMNs in our study.

## 5 Conclusions

In conclusion, histological subtypes of IPMN were significantly associated with radiological type and pathological characteristics including degree of dysplasia, and T stage. The overall survival of patients with gastric IPMNs was much better than that of patients with the pancreatobiliary type IPMNs. Most of the branch-duct IPMNs were gastric type with benign adenoma. However, once the gastric type developed into invasive carcinoma, the overall survival lost its significance with the intestinal and pancreatobiliary types. Therefore, we should pay more attention to the branch-duct IPMNs, especially those in patients who have been followed up according to the Sendai Criteria. In addition, the prognostic value of the histological subtypes on IPMN should be reconsidered.
